# Argan Oil Exerts an Antiatherogenic Effect by Improving Lipids and Susceptibility of LDL to Oxidation in Type 2 Diabetes Patients

**DOI:** 10.1155/2011/747835

**Published:** 2011-11-01

**Authors:** M. M. Ould Mohamedou, K. Zouirech, M. El Messal, M. S. El Kebbaj, A. Chraibi, A. Adlouni

**Affiliations:** ^1^Lipoproteins and Atherosclerosis Research Laboratory, Faculty of Sciences Ben Msik, Casablanca, Morocco; ^2^Laboratory of Biochemistry, Faculty of Sciences Ain Chock, Casablanca, Morocco; ^3^Endocrinology and Nutrition, and Metabolic Diseases Department, University Hospital of Ibn Sina, Rabat, Morocco

## Abstract

In this study, we investigate the effect of argan oil consumption on serum lipids, apolipoproteins (AI and B), CRP, and LDL susceptibility to oxidation in type 2 diabetic patients which are known to have a high level of cardiovascular risk due to lipid abnormalities and lipid peroxidation. For that, 86 type 2 diabetic patients with dyslipidemia were randomized to one group consuming 25 mL/day of argan oil during 3 weeks and control group consuming 20 g/day of butter in breakfast. After argan oil intervention, serum triglycerides decreased by 11.84%, *(P* = 0.001), total chol by 9.13%, *(P* = 0.01), and LDL-chol by 11.81%, *(P* = 0.02). However, HDL-chol and Apo AI increased (10.51%, *P* = 0.01 and 9.40%,  P = 0.045, resp.). Susceptibility of LDL to lipid peroxidation was significantly reduced by increasing of 20.95%, *(P* = 0.038) in lag phase after argan oil consumption. In conclusion, we show for the first time that consumption of argan oil may have an antiatherogenic effect by improving lipids, and the susceptibility of LDL to oxidation in type 2 diabetes patients with dyslipidemia, and can therefore be recommended in the nutritional management of type 2 diabetes.

## 1. Introduction

Increased cardiovascular morbidity and mortality in patients with type 2 diabetes is well established [1]. In Morocco, the diabetes prevalence was 6.6% in 2000, while in 2003 it was progressed to 10% [2]. 

Patients with type 2 diabetes have a high level of cardiovascular risk due to lipid abnormalities and lipid peroxidation which are responsible in the development of atherogenesis. These lipid disorders include not only quantitative but also qualitative abnormalities of lipoproteins. The main quantitative abnormalities are increased triglyceride (TG) levels and decreased HDL-cholesterol (HDL-chol) levels [3, 4]. The main qualitative abnormalities include large very low density lipoprotein (VLDL) particles, relatively rich in triglycerides, small dense low density lipoprotein (LDL) particles, increased triglyceride content of LDL and high density lipoprotein (HDL), glycation of apolipoproteins, and increased susceptibility of LDL to oxidation [5]. The American Diabetes Association (ADA) has set desirable LDL cholesterol (LDL-chol), HDL-chol, and TG levels as <100, >40 in men/>50 in women, and <150 mg/dL, respectively [6]. Apolipoprotein B (Apo B) reflects the total mass of atherogenic particles (VLDL, Intermediate density lipoprotein (IDL), and LDL) and is associated with cardiovascular disease (CVD) independently of LDL-chol levels [7, 8]. In type 2 diabetes patients without other major CVD risk factors, Apo B should be <90 mg/dL. In case of diabetes associated with one or more other cardiovascular risk factors, Apo B should not exceed 80 mg/dL [9]. Some authors have suggested that in type 2 diabetics compared to healthy controls, LDL is more susceptible to oxidation, and diabetic patients have higher levels of oxidation which may contribute to the more aggressive atherosclerosis when compared to nondiabetics [10]. Apolipoprotein AI (Apo AI) is the major component of HDL constituting 45% of its molecular mass and is responsible for its antiatherogenic property by acting as a cofactor for the enzyme lecithin cholesterol acyltransferase (LCAT) and as a mediator in transfer of cholesterol from cells to HDL particles, which are key steps involved in reverse cholesterol transport [11]. It is known that in diabetic patients, the plasma concentration of Apo AI is lower compared with healthy subjects [12]. The Apo B/Apo AI ratio may represent the balance between pro-atherogenic and antiatherogenic lipoproteins [13]. The Apo B/Apo AI ratio has been reported to predict cardiovascular risk better than cholesterol value [14]. The pathophysiology of lipid abnormalities in type 2 diabetes is not yet totally explained. However, insulin resistance and the “relative” insulin deficiency, observed in patients' with type 2 diabetes, are likely playing a crucial role since insulin has an important function in the regulation of lipid metabolism [5]. The C-reactive protein (CRP), an inflammatory marker, is known to play a role in the insulin resistance [15]. Indeed, several studies showed that the association of CRP with insulin resistance was independent of obesity [16].

The diet and lifestyle are an integral part of any diabetes management plan [17]. In the traditional Moroccan diet, argan oil (extracted from *Argania spinosa*, an endemic tree of south-western Morocco) is usually consumed at breakfast, especially in the south-western region of the country. Generally, this oil is rich in unsaturated fatty acids (UFA), principally oleic and linoleic acids (44.8 and 33.7%, resp.) [18]. Interestingly, the unsaponifiable fraction (1% of the oil constituents) of argan oil is mainly rich in antioxidant compounds such as tocopherols, which are present in a higher proportion compared to olive oil (637 mg/kg versus 258 mg/kg, resp.) and especially in its *γ*-isoform (75%) [18]. The quality of fatty acids present in argan oil and their composition in antioxidants give it a special significance in the nutritional prevention of CVD and other diseases [19–25]. Consumption of argan oil could contribute to reduce the risk in patients at high cardiovascular risk. The recent data, published by our team, have shown the beneficial effect of argan oil on lipid profile and LDL susceptibility to lipid peroxidation in healthy subjects and dylipidemic patients. That why we were interested to evaluate its effects in type 2 diabetic patients with dyslipidemia.

## 2. Patients and Methods

### 2.1. Patients

The study protocol was approved by the regional Committee for Ethics. This study was conducted on diabetic patients with dyslipidemia aged 40–80 years consulting the endocrinology department (Ibn Sina university hospital, Rabat, Morocco). Exclusion criteria include presence of hepatic or renal disease, myocardial infarction, cigarette smoking, use of dietary antioxidant supplements, and treatment with insulin, lipid lowering drugs, or hormone therapy during the preceding 6 months. The protocol and objectives of this study were explained to the participants in detail. Among 127 patients, a total of 86 diabetic patients without any diabetes cardiovascular complications (retinopathy, nephropathy, and myocardial infarction) completed the study and 41 dropped out because of personal reasons. During this study, the daily habits of the participants such as physical activities, number of sleeping hours, and working time were not changed. All patients were informed verbally and in writing, and all patients signed an informed consent form before entering the study.

### 2.2. Design

**Figure 1:**
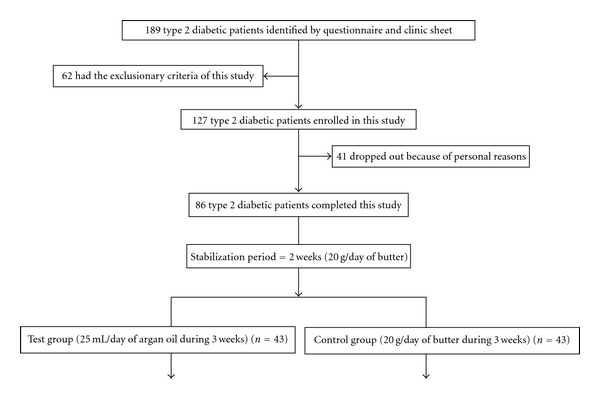
Design of study.

Study design (Figure 1) was two diet periods. In the first diet period for 2 weeks (baseline diet), all the patients consumed 20 g/day of butter with toasted bread for breakfast. In the second diet period for 3 weeks, the patients were randomized to two diet groups: one group of 43 patients consuming 25 mL/day of argan oil (argan oil group) at breakfast, and the second group of 43 patients consuming 25 g/day of butter (control group).

### 2.3. Biological Materials

The argan oil supplied and distributed to the participants was purchased from the same origin and was extracted by industrial process [26]. Its fatty acid and minor components composition is presented in Table 1 [22].

### 2.4. Blood Collection

At the end of each diet period, venous blood was collected into draw tubes after 12 h fast. Plasma was obtained by centrifugation for 12 min for 4000 rpm. Plasma samples were stored at −20°C until analysis.

### 2.5. Food Questionnaire

All of patients have been investigated in relation to their daily eating habits through a comprehensive food questionnaire covering all foods commonly consumed in Morocco and covers all meals and snacks daily.

### 2.6. Measurements

Demographic and anthropometric parameters (age, sex, weight, body mass index (BMI), waist circumference) were evaluated in the patients at baseline. Serum total-chol (TC) and HDL-chol levels were determined by enzymatic colorimetric procedure of Richmond [27] (Randox cholesterol enzymatic kit for cholesterol, and Randox HDL cholesterol precipitant for HDL-C; Crumlin Co., Antrim, UK) adapted for a spectrophotometer (Helios). Serum triglycerides were quantified by an enzymatic colorimetric procedure of Trinder [28]. (Randox, triglycerides enzymatic kit, Crumlin Co, Antrim United Kingdom). In contrast, LDL-chol was obtained by the Friedewald formula [29], that is, LDL-chol = total cholesterol − (triglycerides/5) − HDL-chol. Plasma Apo A1 and Apo B were measured by immunoturbidimetry [30–33] (Cobas Integra 700) with a both inter-assay and intra-assay coefficient of variation of 2% and 1.5% for Apo AI, and 2.1% and 0.66% for Apo B, respectively. The sensitivity is 0.19 mg/dL for Apo AI and 0.052 mg/dL for Apo B. The HbA1c was measured using a commercial kit (Gly-cotest 2; Pierce, Rockford, IL). Serum CRP concentration was measured by immunoturbidimetry with latex particles sensitized with specific antibodies [34–36] (Cobas Integra 700, Roche) with a both inter-assay and intra-assay coefficient of variation of 2.9% and 1.8%, and sensitivity of 0.085 mg/dL. The creatinine was measured by Jaffe kinetic reaction buffer without deproteinization [37–39] with a both inter-assay and intra-assay coefficient of variation of 3.8% and 0.8%, and sensitivity of 0.017 mg/dL. From the plasma, LDL fraction from each sample was isolated by sequential preparative ultracentrifugation using a Beckman ultracentrifuge as described by Sattler et al. [40]. After centrifugation at 100 000 rpm during 2 h at 15°C in a Beckman TLA 100.4 rotor, the LDL fractions were collected with a syringe, and protein composition was measured by commercial assay (Pierce method, Rockford, IL, USA) using bovine serum albumin as a standard. Conjugated diene formation was determined spectrophotometrically by the measure of the absorbance at 234 nm (U-3000 spectrophotometer, Hitachi). Plasma was diluted (1/100) with phosphate buffer before each measure. LDL at concentration of 100 mg/mL underwent in vitro oxidation induced by incubation with 10 Mm CuSO4 at 37°C. The kinetics of conjugated dienes formation was continuously monitored by measuring the absorbance at 234 nm, every 10 min for at least 8 h. The lag phase (LP) of conjugated dienes formation, maximal rate of dienes production (MR), and maximum dienes production was determined according to the method of Kleinveld [41].

### 2.7. Statistical Analysis

Statistical analysis was done using SPSS 17.0 software and using the student test for comparison of two means. The results are expressed as mean ± standard deviation. Difference in LDL susceptibility to lipid peroxidation was analyzed by the Wilcoxon signed rank test. Differences are considered significant when the *P* value is <0.05.

## 3. Results

The baseline characteristics of the patients, including sex and age as well as the risk factors associated with atherosclerosis diseases, are detailed in Table 2. Average glycemic control was poor (>7%). Indeed, the mean of HbA1c in both argan oil and control group was 8.8% and 9%, respectively. According to the International Diabetes Federation definition (IDF) [42], the metabolic syndrome (MS) percentage in both groups of argan oil and control was 49.34% and 51.15%, respectively. The most frequent components of the metabolic syndrome in our diabetic patients in addition to hyperglycemia were central obesity and high level of plasma triglycerides. Total of the patients had at least two MS components. In argan oil group, 40% of patients had tree of the five IDF criteria, 50% had four, and 10% had the five MS components. In control group, 30% of patients had tree of the five IDF criteria, 66% had four, and 4% had the five MS components. The Apo B/Apo AI ratio is strongly associated with the presence of individual metabolic syndrome components, with the metabolic syndrome itself, and with insulin resistance. The Apo B/Apo AI ratio was elevated in both argan oil and control group at baseline (0.65 and 0.66, resp.).

The analysis of diet questionnaire showed that 92% of patients consume at least once a day fruits and vegetables, 55% consume at least once a day cereals and legumes. However, only 50% consume meat, tea, and coffee at least once a week, whereas no tendency to consume oils, fats, and sugar was noted.

**Figure 2:**
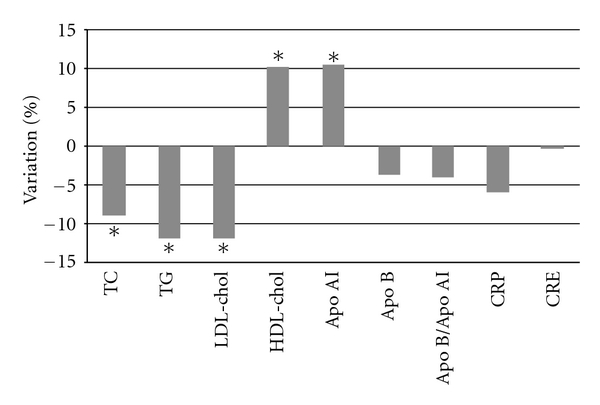
Variations of different parameters in argan oil groups after three weeks of interventions. Abbreviations: Apo AI: apolipoprotein AI; Apo B: apolipoprotein B; CRE: creatinine; CRP: C-reactive protein; HDL-Chol: high density lipoprotein cholesterol; LDL-chol: low density lipoprotein cholesterol; TC: total cholesterol; TG: triglycerides; *significantly different.

The results obtained after three weeks of intervention with argan oil showed a significant change in lipid parameter and apolipoprotein AI (Table 3). The change in CRP and Apolipoprotein B was not significant but was a tendency of reduction (a decrease of 5.85% and 3.65%, resp.) (Figure 2). Serum triglycerides decreased by 11.84%, (*P* = 0.001), total cholesterol by 9.13%, (*P* = 0.01), and LDL-chol by 11.81% (*P* = 0.02). However, a significant increase was observed in HDL-chol and Apo AI plasma concentration (10.51%, *P* = 0.01 and 9.40%, *P* = 0.045, resp.). Susceptibility of LDL to lipid peroxidation was significantly decreased by increasing of the lag phase (20.95%, *P* = 0.027) after 3 weeks of argan oil intervention. The MR and MDP were significantly decreased (Table 4).

## 4. Discussion

The quality of fatty acids contained in argan oil and the abundance of antioxidant compounds in the unsaponifiable fraction suggests a putative use for this oil in nutritional prevention against some pathologies such as cardiovascular diseases (CVD). However, only few studies have reported beneficial pharmacological effects of argan oil and its compounds in blood pressure, lipids and lipids peroxidation in human and animal models [19–23]. This is probably related to its fat composition, 45% monounsaturated fatty acids (MUFA) and 35% polyunsaturated fatty acids (PUFA), and to its minor compounds such as polyphenols, phytosterols, and tocopherols [18]. The hypolipidemic and the antioxidant effects of argan oil were demonstrated in healthy subject [22, 23]. Another study showed that intake of argan oil, improves insulin signaling in fat and liver beyond levels found in a rat model of dietary-induced obesity [43]. 

The lipid lowering effect of argan oil was investigated for the first time in diabetic patients, in this study and it could be expected because of the interesting level of unsaturated fats and the ratio of PUFA/SFA (3.34) and of MUFA/PUFA (1.63) [20]. PUFA has protective effects against oxidation, as explained by the presence of the double bonds. Argan oil is rich in oleic and linoleic acids that show less susceptibility to peroxidation [20]. Moreover, linoleic acid derivatives, particularly gamma-linolenic acid are shown potent in reducing blood cholesterol in humans and rats [44–46]. 

Also, argan oil is rich in phytosterols known for their beneficial effects on lipid markers [47–51]. The hypocholesterolemic effect of argan oil may be due to its high content in sterols in the minor compounds fraction. A recent study shows that plant sterols in patients with hypercholesterolemia reduce the concentration of cholesterol in both LDL and small LDL which are considered the more atherogenic particles [52, 53].

It is well documented that Apo AI is inversely correlated to CVD risk as to HDL-chol level. Apo AI as HDL-chol levels have significantly increased after argan oil intervention assuming that argan oil is antiatherogenic oil and could be used as nutritional preventive oil.

The diabetic patients in this study present a tendency to decrease the Apo B/Apo AI ratio after argan oil intervention, while they have showed an elevated Apo B/Apo AI ratio at baseline as in patients with metabolic syndrome, coronary artery disease, and with ischemic stroke [54, 55].

The interesting finding of this study was the combination of the decrease of triglycerides and Apo B/Apo AI ratio and the increase of HDL-chol, obtained after argan oil consumption which could reduce the percentage of metabolic syndrome observed among the patients, thus giving a beneficial role in the management of metabolic complications associated with metabolic syndrome in type 2 diabetics.

At the end of intervention period, we observed an important and significant increase of lag phase of conjugated dienes formation in LDL oxidation. This may be explained by the fact that isolated LDL particles from patients might be enriched with different antioxidants from argan oil diets, reducing their susceptibility to lipid peroxidation [56, 57]. Drissi et al. [22] showed that sterol, tocopherol, and phenol compounds from argan oil increase the resistance of LDL to oxidation in healthy subjects. This study brings a new demonstration of the antioxidant effect of argan oil.

The trend of reduction of CRP is important considering the improve of insulin sensitivity in target tissue and may increase the ability of insulin to induce its beneficial effect on lipid metabolism.

## 5. Conclusion

Our findings show for the first time that consumption of argan oil may have an antiatherogenic effect by improving lipids and apolipoprotein AI and the susceptibility of LDL to oxidation in type 2 diabetes patients with dyslipidemia and can therefore be recommended in the nutritional management of type 2 diabetes.

## Figures and Tables

**Table 1:** Chemical composition of argan oil [22].

Fatty acids	%
C 16 : 0	13.4
C 18 : 0	5.1
C 18 : 1	44.8
C 18 : 2	35.7
C 18 : 3	0.1

Sterols	mg/100 g oil

Schottenol	142
Spinasterol	115
Stigmasta-8,22-dien-3*β*-ol	9
Other	29

Tocopherols	mg/Kg oil

*α*	35
*β*	122
*γ*	480

Phenolics compound	*μ*g/Kg oil

Vanilic acid	67
Syringic acid	37
Ferulic acid	3147
Tyrosol	12

**Table 2:** Baseline anthropometrical and biological characteristics of both study groups.

Characteristics	Argan oil group	Control group
M/W	23/20	22/21
Age (years)	52.09 ± 10.75	52.29 ± 10.51
Diabetes (years)	7.63 ± 4.34	7.71 ± 4.8
Family history (%)	19.56	15.38
SBP (mmHg)	12.00 ± 2.42	12.77 ± 2.43
DBP (mmHg)	7.67 ± 1.28	7.74 ± 1.34
Hypertension (%)	26.08	23.03
Weight (kg)	75.61 ± 13.08	76.03 ± 13.25
BMI (kg/m^2^)	29.67 ± 5.00	29.71 ± 4.90
Overweight (%)	26.08	25.64
Obesity (%)	19.57	21.76
Waist circumference (cm)	100.79 ± 15.46	102 ± 14.92
Metabolic syndrome according to IDF definition %	49.34	51.84
TC (mg/dL)	197 ± 35	191 ± 37
TG (mg/dL)	152 ± 55	150 ± 56
HDL-chol (mg/dL)	39 ± 7	34 ± 5
LDL-chol (mg/dL)	127 ± 39	127 ± 33
Apo B (mg/dL)	82.90 ± 20.13	86.83
Apo AI (mg/dL)	106.84 ± 17	106.24 ± 14
Apo B/Apo AI	0.65	0.66
HbA1C %	8.8 ± 1	9 ± 0.7
CRP (mg/dL)	5.22 ± 1.7	5.34 ± 1.4
Creatinine (mg/dL)	6.1 ± 1.16	6.07 ± 1.2
Lag time (min)	52.70	53
MR (mol diene/mol LDL/min)	3.83	3.86
MDP (mol diene/mol LDL)	520.17	521.80

Abbreviations: Apo AI: apolipoprotein AI; Apo B: apolipoprotein B; BMI: body mass index; CRP: C-reactive protein; DBP: diastolic blood pressure; HDL-chol: high density lipoprotein cholesterol; IDF: International Diabetes Federation; LDL-chol: low density lipoprotein cholesterol; M: men; MDP: maximum diene production; MR: maximal rate; TC: total cholesterol; TG: triglycerides; W: women.

**Table 3:** Variations of different parameters in both groups after three weeks of intervention.

Groups		Parameters								
		TC (mg/dL)	TG (mg/dL)	LDL-chol (mg/dL)	HDL-chol (mg/dL)	Apo AI (mg/dL)	Apo B (mg/dL)	ApoB/Apo AI	CRP (mg/dL)	CRE (mg/dL)
Control	After stabilization (2 weeks)	191 ± 37	150 ± 56	127 ± 33	34 ± 5	106.24 ± 14	86.83 ± 20.63	0.66 ± 0.10	3.96 ± 1.4	6.07 ± 1.2
	After intervention (3 weeks)	192 ± 35	148 ± 54	127 ± 34	35 ± 5	105.71 ± 17	88.11 ± 13.02	0.67 ± 0.17	4.0 ± 1.5	6.21 ± 1.4
	*P*	0.331	0.367	0.451	0.329	0.358	0.462	0.405	0.427	0.391

Argan oil	After stabilization (2 weeks)	197 ± 35	152 ± 55	127 ± 39	39 ± 7	106.84 ± 17	82.90 ± 20.13	0.65 ± 0.13	4.27 ± 1.7	4.65 ± 1.16
	After intervention (3 weeks)	179 ± 36	134 ± 43	112 ± 33	43 ± 10	117.52 ± 10	79.00 ± 14.08	0.63 ± 0.12	4.02 ± 1.34	4.63 ± 1.36
	*P*	0.012	0.001	0.024	0.013	0.017	0.147	0.092	0.075	0.316

Abbreviations: Apo AI: apolipoprotein AI; Apo B: apolipoprotein B; CRE: creatinine; CRP: C-reactive protein; HDL-chol: high density lipoprotein cholesterol; LDL-chol: low density lipoprotein cholesterol; TC: total cholesterol; TG: triglycerides. Values are mean ± SD.

**Table 4:** Kinetic of conjugated dienes formation from oxidized LDL of both study groups after 3 weeks of argan oil intervention.

Groups		Markers of susceptibility of LDL to lipid peroxidation		
		LP (min)	MR (mol diene/mol LDL/min)	MDP (mol diene/mol LDL)
Control	After stabilization (2 weeks)	53.45	3.83	520.17
	After intervention (3 weeks)	52.70	3.86	521.80
	*P*	0.189	0.207	0.180

Argan oil	After stabilization (2 weeks)	52.00	3.88	522.70
	After intervention (3 weeks)	63.10	1.04	338.60
	*P*	0.027	0.031	0.019

Abbreviations: LP: lag phase; MDP: maximum dienes production; MR: maximal rate of dienes production. Values are means ± SD.
